# Permeation of Therapeutic Drugs in Different Formulations across the Airway Epithelium In Vitro

**DOI:** 10.1371/journal.pone.0135690

**Published:** 2015-08-14

**Authors:** Claudia Meindl, Sandra Stranzinger, Neira Dzidic, Sharareh Salar-Behzadi, Stefan Mohr, Andreas Zimmer, Eleonore Fröhlich

**Affiliations:** 1 Center for Medical Research, Medical University of Graz, Graz, Austria; 2 Research Center Pharmaceutical Engineering GmbH, Graz, Austria; 3 Department of Pharmaceutical Technology, Institute of Pharmaceutical Sciences, Karl-Franzens-University of Graz, Graz, Austria; University of Alabama at Birmingham, UNITED STATES

## Abstract

**Background:**

Pulmonary drug delivery is characterized by short onset times of the effects and an increased therapeutic ratio compared to oral drug delivery. This delivery route can be used for local as well as for systemic absorption applying drugs as single substance or as a fixed dose combination. Drugs can be delivered as nebulized aerosols or as dry powders. A screening system able to mimic delivery by the different devices might help to assess the drug effect in the different formulations and to identify potential interference between drugs in fixed dose combinations. The present study evaluates manual devices used in animal studies for their suitability for cellular studies.

**Methods:**

Calu-3 cells were cultured submersed and in air-liquid interface culture and characterized regarding mucus production and transepithelial electrical resistance. The influence of pore size and material of the transwell membranes and of the duration of air-liquid interface culture was assessed. Compounds were applied in solution and as aerosols generated by MicroSprayer IA-1C Aerosolizer or by DP-4 Dry Powder Insufflator using fluorescein and rhodamine 123 as model compounds. Budesonide and formoterol, singly and in combination, served as examples for drugs relevant in pulmonary delivery.

**Results and Conclusions:**

Membrane material and duration of air-liquid interface culture had no marked effect on mucus production and tightness of the cell monolayer. Co-application of budesonide and formoterol, applied in solution or as aerosol, increased permeation of formoterol across cells in air-liquid interface culture. Problems with the DP-4 Dry Powder Insufflator included compound-specific delivery rates and influence on the tightness of the cell monolayer. These problems were not encountered with the MicroSprayer IA-1C Aerosolizer. The combination of Calu-3 cells and manual aerosol generation devices appears suitable to identify interactions of drugs in fixed drug combination products on permeation.

## Introduction

Inhaled medicines compared to oral medication have the advantage of shorter onset times and increased therapeutic ratio due to reduced first pass effect and higher permeation across the epithelium [1]. Active pharmaceutical ingredients (APIs) can be delivered to the lungs by nebulizers, metered dose inhalers and dry powder inhalers, as single compounds or in a fixed dose combination [2]. Currently, most formulations are designed for local therapy of common respiratory diseases, such as asthma, chronic obstructive pulmonary disease (COPD), and cystic fibrosis (CF). However, systemic delivery of insulin has already entered the market, Afrezza is available for treatment of diabetes mellitus types I and II, and several types of molecules and peptides are in preclinical development.

For the development of new formulations it would be helpful to estimate permeation, metabolism and potential drug interference at the epithelium by in vitro screening systems. A recent study showed that the presence of salmeterol decreased the transport of fluticasone across Calu-3 cells [3], suggesting that interaction at the cellular level could partly explain the variable efficacy of fixed dose combinations in clinical trials compared to the same doses applied by single inhalers.

Permeation of drugs for oral delivery is assessed by application of the dissolved APIs to Caco-2 monolayers. Other types of cells, usually cell lines are used for pulmonal drug delivery [4]. For physiologically representative exposure, these cells are cultured at an air-liquid interface where cells are supplied with medium only from the basolateral side while the apical side is exposed to air. Calu-3 cells are most commonly used for the assessment of pulmonary permeability because they reached higher TEER values than measured in the rabbit airways and showed good correlation of permeability values with drug absorption from the rat lung in vivo [5–8]. Furthermore, drug release from disodium cromoglycate (DSCG)/polyvinyl alcohol (DSCG/PVA) microparticles in Calu-3 cells showed good correlation to suitable models [9]. Calu-3 cells are good models because they show similar transporter expression to respiratory cells [10,11]; protein expression of human lung tissue is highest for the organic cation transporter OCTN1, followed by multidrug resistance protein 1 (MRP1), breast cancer resistance protein 1 (BCRP1), ATP-binding cassette sub-family G member 2 (ABCG2), and organic anion transporter OATP2B1. Many inhaled compounds, for instance beclomethasone, budesonide, flunisolide, fluticasone, mometasone, salbutamol, and ipratropium, interfere with efflux pumps (MDR1/P-glycoprotein, MRP1, etc.) and organic cation transporters (OCT1-3, OCTN1, and OCTN2). OCTN1 and OCTN2 are expressed at high levels, OCT1 and OCT3 at moderate levels, and OCT2 at low levels in whole lung tissue [12]. Expression of MDR1/P-glycoprotein, OCTN1, OCTN2, PEPT2 was specifically documented in alveolar epithelial cells [6]. Calu-3 cells express OCT1, OCT3, OCTN1 and OCTN2 and peptide PEPT1/PEPT2 transporters, as well as MDR1/P-glycoprotein [13–15]. All proteins have been detected in Calu-3 cells in submersed culture and in air-liquid interface culture [13,16].

In order to mimic realistic transport and toxicity studies, air-liquid interface culture should be combined with application of aerosols [17]. A variety of elaborate exposure systems have been described for cellular exposure to nebulized and dry aerosols, for instance CULTEX [18,19] and ALICE CLOUD system [20]. However, these systems need specialized equipment and know-how, may lead to particle-dependent differences in deposition, and cannot easily be adapted to the different forms of aerosol applications [21]. Animal studies are the gold standard in the preclinical evaluation of pharmaceutical formulations and intratracheal instillation is the most commonly used application method because delivered doses by whole body exposure or nose-only inhalation vary due to differences in breathing, deposition on the furs, and oral uptake by licking [22]. The use of the same delivery devices as for animal experimentation might allow a better comparison of in vitro data and results obtained in animals because particles that are being delivered are of comparable sizes in both exposures. High local drug concentrations are a problem but occur also in intratracheal instillation [23]. MicroSprayer IA-1C Aerosolizer for liquid aerosols and DP-4 Dry Powder Insufflator for powder aerosols have been used for intratracheal delivery of nanoparticles and toxicants to mice and rats [24–27]. The MicroSprayer has also been used in cellular studies [21,28–36].

In order to find out whether these devices could be used in cellular experiments we compared different exposure models based on Calu-3 cells regarding transport of APIs in different aerosol formulations. Sodium fluorescein was chosen as paracellular transport marker with no distinct directionality while rhodamine 123 served to assess the effect of the MDR1/P-glycoprotein efflux pump. As examples for relevant drugs, budesonide and formoterol fumarate were chosen. The glucocorticoid budesonide and the long-acting beta-adrenoreceptor agonist formoterol fumarate are routinely used drugs in the treatment of obstructive lung diseases. Budesonide as well as formoterol fumarate are sold as single drugs in various pressurized metered dose inhalers and dry powder inhalers. The fixed dose combination product is marketed by AstraZeneca under the trade name Symbicort.

## Materials and Methods

### Compounds

Sodium fluorescein, rhodamine 123, and budesonide were purchased from Sigma-Aldrich (Vienna, Austria) and formoterol fumarate dihydrate from Eubio (Vienna, Austria). Symbicort 160/4.5 Turbuhaler (AstraZeneca GmbH, Vienna, Austria) powder was removed from a commercially available device. Stock solutions were prepared with DMSO (43 mg/ml budesonide, 20 mg/ml formoterol fumarate, 4 mg/ml Symbicort 160/4.5) and diluted in Krebs-Ringer buffer.

### Cell culture

Calu-3 cells were obtained from the American Type Culture Collection (ATCC, HTB-55, LGC Standards GmbH, Wesel, Germany). Cells were cultured in 90% Minimum Essential Medium (MEM) with Earle’s salts, 10% fetal bovine serum, 2 mM L-glutamine and 1% penicillin-streptomycin (PAA Laboratories and Lifetechnologies, Vienna, Austria) at 37°C in humidified air atmosphere containing 5% CO_2_ in 175 cm^2^ cell culture flasks (Greiner Bio-One GmbH, Rainbach, Austria).

0.5 x 10^6^ cells (passage 32–38) were seeded per 12-well Transwell insert. To study the influence of material properties on the system, membranes made from different materials (polycarbonate, polyester, polyethylene terephthalate) in pore sizes of 0.4 μm and 3 μm obtained from Corning (Szabo-Scandic, Vienna, Austria) and Greiner Bio-One GmbH, were used. Cells were cultured in submersed conditions with 500 μl medium in the apical compartment and 1500 μl in the basolateral compartment. Medium was changed every 2 or 3 days. For air-liquid interface culture, the cell culture medium was removed either one day post-seeding or for 3 days after the cells had reached a transepithelial electrical resistance value >700 Ω*cm^2^ in submersed culture. In air-liquid interface culture, medium (600 μl) in the basolateral compartment was changed every 2–3 days. For safety reasons, exposures with the compounds were performed in a HERAsafe KS 9 clean bench (Thermo Scientific, Vienna, Austria) equipped with UPLA filters of both filter grades U15 and H14.

### Transepithelial electrical resistance (TEER)

TEER values were determined for all cultures every 2–3 days with an EVOM STX-2-electrode (World Precision Instruments, Berlin, Germany). 0.5 ml MEM were added to the apical and 1.5 ml MEM to the basolateral compartment for TEER measurements. Prior to the reading cells were equilibrated for 30 min in the incubator. TEER values were calculated as follows:

$$\text{TEER}\;(\Omega \;*\;{\text{cm}}^{2})=(\text{Sample}–\text{blank resistance},\;\text{given in}\;\Omega )\;*\;\text{membrane area},\;\text{given in}\;{\text{cm}}^{2}$$

Blank resistance is defined as the resistance of the membrane without cells and the membrane area for 12-well inserts is calculated to be 1.12 cm^2^/well. TEER values were also determined at the beginning and the end of the transport studies using Krebs-Ringer buffer instead of medium. When Krebs-Ringer buffer was used TEER values were ~ 80 Ω*cm^2^ lower than for cell culture medium.

### Cytotoxicity by formazan bioreduction

Bioreduction to formazan was used as an indication for viable monolayers. CellTiter 96 Aqueous Non-Radioactive Cell Proliferation Assay (Promega, Mannheim, Germany) was used according to the manufacturer’s instructions. To all wells the combined MTS/PMS solution (200 μl + 1 ml medium) was added. Plates were incubated for 2 hours at 37°C in the cell incubator. Absorbance was read at 490 nm on a plate reader (SPECTRA MAX plus 384, Molecular Devices, Biberach, Germany).

### Membrane damage according to lactate dehydrogenase release

The CytoTox-ONE Homogeneous Membrane Integrity Assay (Promega, Mannheim, Germany) was used according to the instructions given by the producer and fluorescence was recorded with an excitation wavelength of 544 nm and an emission wavelength of 590 nm. After subtraction of the blank value the average fluorescence from the samples was normalized to the maximum LDH release (lysis control).

### Transport studies

0.5 x 10^6^ Calu-3 cells (passage 32–38) were seeded per membrane and different exposure systems were used (Fig A in S1 File). To discriminate between the different exposures, the following terminology is used. Cultures with cells supplied with medium only from the basolateral side are termed air-liquid interface culture. In submersed liquid exposure (SLE) cells are cultured in submersion and exposed with compound dissolved in buffer. In the air liquid exposure (ALE) cells are cultured at an air-liquid interface and the compound dissolved in buffer is applied to the cells. For air-liquid interface deposition (AID) cells are cultured at an air-liquid interface and exposed to the compound applied as liquid aerosols by MicroSprayer IA-1C Aerosolizer (AID_M) or as powder aerosols by DP-4 Dry Powder Insufflator (AID_D). For comparison of the different exposure systems Calu-3 cells cultured for 11 days on 3 μm 12-well insert (Greiner Bio-One) were used.

Compounds were applied in Krebs-Ringer buffer (142 mM NaCl, 3 mM KCl, 1.2 mM MgCl_2_, 1.5 mM K_2_H_4_PO_4_, 4.2 mM CaCl_2_, 25 mM NaHCO_3_, 4 mM glucose, 10 mM Hepes, pH 7.4). For SLE and ALE exposures the apical compartment was filled with 500 μL and the basolateral compartment with 1500 μL. Concentrations were 10 μg/ml (26 μM) fluorescein sodium, 100 μg/ml rhodamine 123 (262 μM), 100 μM budesonide and 1 mM formoterol fumarate. For AID studies the basal compartment was filled with 700 μl, and 200 μl of drug dissolved in Krebs-Ringer buffer were sprayed on each Transwell insert using the MicroSprayer IA-1C Aerosolizer (AID_M exposure). 1 mg (2.66 μmol) fluorescein sodium, 1 mg (2.63 μmol) rhodamine 123, 1 mg (2.3 μmol) of budesonide, 1 mg (2.9 μmol) formoterol fumarate as single compound or 1 mg Symbicort 160/4.5 Turbuhaler powder (AstraZeneca GmbH) consisting of 0.46 μmol budesonide and 0.017 μmol formoterol were sprayed on the Transwell Inserts using the DP-4 Dry Powder Insufflator (AID_D exposure). To identify cytotoxicity by high compound concentrations in the exposures with DP-4 Dry Powder Insufflator, 100 μl of a suspension (1 mg in 200 μl Krebs-Ringer buffer) of the respective compounds was applied to the cells. The dose was chosen based on delivery rates in animals [37] with the limitation that no complete dissolution could be obtained. Viability assessed by MTS assay (cytotoxicity) did not show a significant decrease of viability, release of LDH or significant decrease of TEER values after 2h (data not shown). 100 μl samples from the basolateral compartment were taken at 0–30–60–90–120 min and replaced with pre-warmed Krebs-Ringer buffer. Well plates were shaken using an orbital shaker at stirring rate 200 rpm and 37°C for the entire exposure time. At the end of the SLE and ALE studies the content of the basolateral compartment and 10 μl of the apical compartment were collected. After the AID studies 100 μl of Krebs-Ringer buffer for the fluorescent dyes and 100 μl DMSO for budesonide and formoterol was added to the apical chamber. The total volume of this solution was determined and this value minus the added 100 μl used for calculations of drug concentrations. The entire content of the basolateral compartment and 10 μl of the apical compartment were collected. In the initial experiments, after the last sample was taken, cells were lysed with 20 μL Triton-X 100 and 70% ethanol (50:50) for 30 min under agitation using an orbital shaker. The contents of the basolateral and the apical compartments were pooled, filtered and either determined immediately by fluorescent detection or stored at -20°C in HPLC vials until measurement by HPLC. Lysis was omitted in subsequent experiments because the amount of budesonide in the cells was very low (0.96 μg) related to the total recovered amount of 19.3±0.7 μg. It was not expected that these amount would strongly affect P_app_ values, which are based on the time dependency of the transport across the monolayer.

For the determination of the permeability coefficient (P_app_) the following equation, where dQ/dt is the flux across the cell monolayer (ng/sec), A the surface of the monolayer (cm^2^) and C the initial concentration in the apical compartment (ng/ml), was used:

$${\text{P}}_{\text{app}}=\frac{\text{dQ}}{\text{dt}\;*\;\text{A}\;*\;\text{c}}$$

In the case of AID where the drug was applied as powder no P_app_ values were calculated because of non-linear transport of the compounds and unknown initial volume in the apical compartment.

### Application devices

The MicroSprayer IA-1C Aerosolizer (PennCentury Inc., Wyndmoor, PA) was used to mimic nebulization of the compounds. The device consists of a thin, flexible, stainless steel tube measuring 0.64 mm in diameter and 50.8 cm in length attached to the light, hand-operated, high-pressure syringe FMJ-250. A unique patented atomizer at the very tip of the tube generates the aerosol with a mass median diameter of 16–22 μm (http://www.penncentury.com/products/IA_1C.php). The device was fixed at a distance of 11 cm between tip of the MicroSprayer and the rim of the exposure plate. Inserts were transferred to a separate (exposure) plate to avoid contamination of the adjacent inserts and 200 μl of the aerosol were applied per well (Fig B, a in S1 File). After the treatment the insert was replaced into the original plate for transport studies. The device was cleaned with 70% ethanol and aqua dest. at the end of the experiments and dried at 60°C overnight.

The DP-4 Dry Powder Insufflator (PennCentury Inc., Wyndmoor, PA) was used to simulate the deposition by a dry powder inhaler and was fixed at a distance of 3–10 cm between tip of the tube and the rim of the exposure plate. Two delivery tube lengths were tested; one 52.3 cm and the other 7.3 cm long. In contrast to the delivery tube used for in vivo application, the delivery tube had a straight form. The shorter delivery tube produced more reproducible results and was used for the subsequent experiments (Fig B, b in S1 File). Inserts were transferred to a separate plate to avoid contamination of the adjacent inserts and 1 mg of the powder weighted into the sample chamber was applied. After the treatment the insert was returned to the original plate. In pilot experiments the influence of the distance between the end of the delivery tube and the cells as well as the role of repeated actuation of the syringe was studied. Damage of monolayer integrity was interpreted as difference between TEER values before and after the experiments > 100 Ω*cm^2^. For the experiments the set-up with shorter tube and longer distance between tip and cells was chosen. Between the applications the device was cleaned with a compressed air cleaner spray and in addition by piercing with a fine wire. After the experiments it was cleaned with 70% ethanol and aqua dest. in addition and dried at 60°C overnight.

Particles that were delivered by the DP-4 Dry Powder Insufflator were analyzed by bright-field microscopy. The particles were applied to a glass slide instead of an insert and viewed at an upright microscope (BX-51, Olympus, Vienna, Austria) at magnification x10 to demonstrate the particle distribution pattern and at x40 to determine particle sizes. Sizes were measured using Cell^D Imaging software (Olympus).

### Quantification

Fluorescent samples were detected at excitation wavelength of 485 nm and emission wavelength of 520 nm for sodium fluorescein and rhodamine 123. Samples were diluted with Krebs-Ringer buffer and measured with a FLUOstar Optima (BMG Labtech, Graz, Austria). Linearity was proven for 0.12–10 μg/ml sodium fluorescein and for 1.25–100 μg/ml rhodamine 123.

Budesonide and formoterol fumarate were quantified by high-performance liquid chromatography electro spray ionization mass spectrometry (HPLC-ESI-MS) using an Acquity UPLC H-Class system (Waters, Vienna, Austria) equipped with a photodiode array detector and a single quadrupole detector. As stationary phase a Superspher 100 RP-18e column (125mm x 4mm i.d, 4 μm particle size) was used. Mobile phase was composed of acetonitrile (A) and 10 mM ammonium acetate buffer pH 3.0 (B) using the following gradient program: 2% A (0–0.5 min), 2–95% A (0.5–6.0 min), 2% A (6.1–10.0 min).) At a flow rate of 0.6ml/min, retention times were 7.1 min and 5.6 min for budesonide and formoterol, respectively. Mass spectrometric detection was operated in single ion recording (SIR) mode using m/z 345.1 for formoterol and m/z 431.2 for budesonide. Capillary voltage was set to 0.5 kV and 2.5 kV and cone voltage to 25 V and 20 V, for formoterol and budesonide respectively. The limit of detection for both, budesonide and formoterol fumarate was determined with 20 ng/ml; linearity was proven for budesonide and formoterol fumarate in the range of 20 ng/ml to 500 ng/ml. Krebs-Ringer buffer did not interfere with the measurements.

### Immunocytochemistry

For detection of mucus production, the membranes were excised from the plastic insets and washed 3 x 5 min with PBS. Subsequently, membranes were fixed with 4% paraformaldehyde for 20 min at RT and washed again 3 x 5 min with PBS. After permeabilization with PBS containing 0.2% Triton X-100 for 2h at RT, blocking with 10% normal goat serum (Zymed Medical Product GmbH, Vienna, Austria) for 30 min at RT, incubation with mouse anti-mucin 5AC ([45M1], Abcam, 1:200, Cambridge, UK) or normal mouse Immunglobulin G (DAKO diagnostic, Hamburg, Germany) for negative controls was performed at 37°C for 60 min followed by goat Alexa Fluor 488-labelled anti-mouse IgG antibody (1:400, Life technologies, Vienna, Austria) again at 37°C for 60 min. Nuclei were counterstained with 1 μg/ml Hoechst 33342 (Life technologies) for 15 min at RT. Incubations were performed under light protection and between the antibody incubations the membranes were rinsed 3 x 5 min in PBS. Subsequently, all membranes were mounted in fluorescence mounting medium from DAKO and stored in the dark. Cells were viewed at a 510 LSM Meta (Zeiss, Vienna, Austria) using excitation at 405 nm and detection with a BP 420–480 nm filter for the nuclear stain. Binding of the anti-mucin antibody was recorded at excitation at 488 nm and detection with a BP 505–550 nm filter.

For quantification of mucus production after different duration of air-liquid interface culture images of anti-mucin 5AC staining were taken with the same settings at the LSM510 Meta and mean intensities in the different channels (blue, green) determined using Image J 1.49v software. For each condition, 11 days and 3 days of air-liquid interface culture and antibody negative control, 800 cells were evaluated. Representative images that were analyzed are shown in Fig C in S1 File.

### Statistics

Data from ≥ three independent experiments were subjected to statistical analysis. These values are represented as means ± S.D. and have been analyzed with a one-way analysis of variance (ANOVA), followed by a Tukey-HSD post hoc test for multiple comparisons (IBM SPSS statistics 19 software). Independent t-test and Levine’s Test for equality of variances analyzed the differences between two mean values. The results with p-values of less than 0.05 were considered to be statistically significant.

## Results

To allow the comparison of our data with the Calu-3 models used in other studies the influence of the duration of the air-liquid interface culture and of different membranes was determined (Table 1). Characteristics of the Transwell inserts from Corning and Greiner Bio-One (product information) are provided in S1 Table. Aerosols generated by MicroSprayer IA-1C Aerosolizer and DP-4 Dry Powder Insufflator were assessed for deposition rate, effects on the Calu-3 monolayer, and particle size (dry powder).

**Table 1:** Influence of culture conditions on TEER values, P_app_ values for fluorescein and time until TEER was reached (n = 12).

Parameter	Condition		
	Submersed	Air-liquid interface	
	3 μm pores	0.4 μm pores	3 μm pores
TEER (Ω*cm^2^)	741±101	484±152	538±105
Time until TEER ALI >450 Ω*cm^2^, submersed > 650 Ω*cm^2^ (days)	7±2	14±4	11±2
P_app_ values for fluorescein (x10^-6^ cm/s)	0.09±0.04	0.23±0.04	0.27±0.08

Abbreviation: ALI, air-liquid interface

### Calu-3 cells

**Fig 1:**
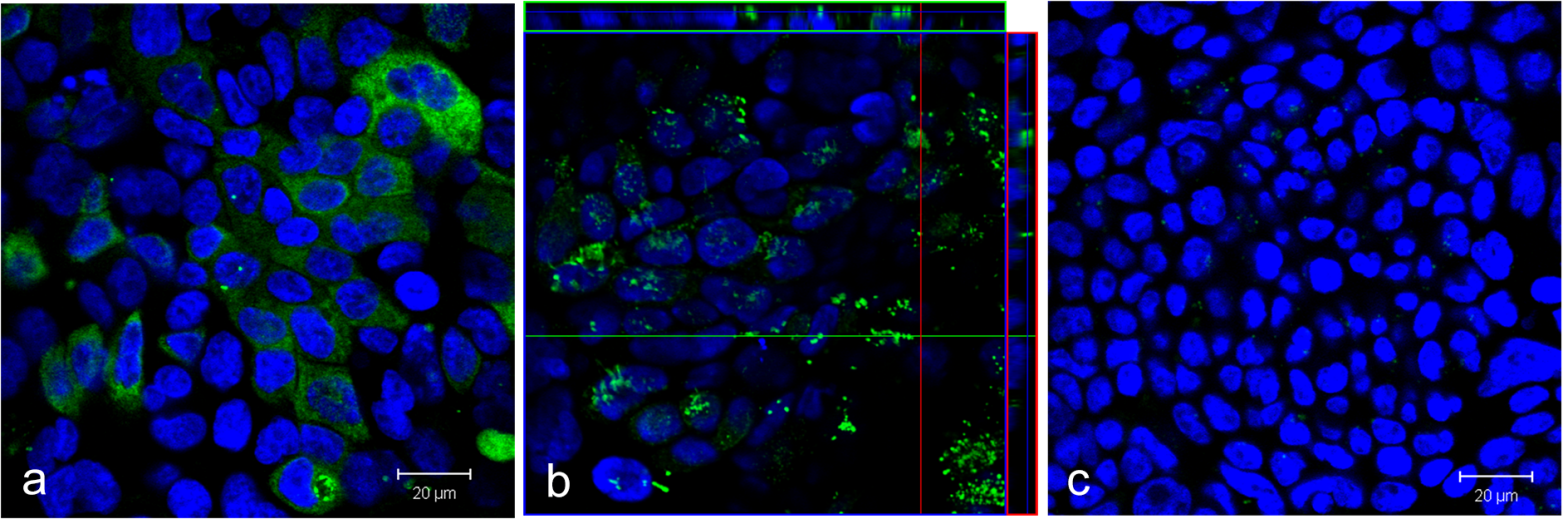
Immunocytochemical detection of mucin 5AC in Calu-3 cells cultured at an air-liquid interface (green, a). Radial sections of z-stacks show that reactivity is located at the apical part of the cells (b). Calu-3 cells cultured in submersion (c) do not show staining against mucin 5AC. Nuclei are stained in blue. Scale bar: 20 μm.

TEER values were significantly higher in cells cultured under submersed conditions (741 ± 101 Ω*cm^2^) than under air-liquid interface condition (mean of cultures in different air-liquid interface conditions and on different membranes, 494 ± 119 Ω*cm^2^). There was no difference in TEER values and in P_app_ values for fluorescein between Calu-3 cells cultured on membranes with 0.4 μm and 3 μm pores but Calu-3 cells on membranes with 0.4 μm pores required a slightly longer time to reach the required TEER values (Table 1). Since the time to reach these TEER values was also more variable for 0.4 μm, in the transport studies membranes with 3 μm pore size were used. TEER values were not influenced by differences in membrane materials (not shown), and different protocols of air-liquid interface culture (3 versus 11 days prior to the experiments) did not result in significantly different TEER values (538±105 vs. 461±99 Ω*cm^2^). Mucin production after submersed culture and culture at an air-liquid interface for 11 days and for 3 days was visualized by anti-mucin5AC staining. Secretory granules located at the apical side of Calu-3 cells were seen in air-liquid interface culture (Fig 1A and 1B) but not in Calu-3 cells in submersed culture (Fig 1C). Tile scans of confocal images showed considerable differences in the intensity of the anti-mucinAC staining between cells but image analysis of anti-mucin 5AC stained cells showed no differences in mean intensities between cells cultured at an air-liquid interface for 3 days versus 11 days prior to the experiments Fig D in S1 File). Means (RFU) in the green channel were 3629.88 ± 691.92 for 11 days air-liquid interface culture vs. 3695.33 ± 501.51 for 3 days air-liquid interface culture. Negative controls (background) showed an intensity of 0.28 ± 0.01. When normalized to intensity of the nuclear stain (blue channel) ratios of 0.54 ± 0.04, 0.53 ± 0.18, and 0.00 ± 0.00 were obtained for 11 days air-liquid interface culture, 3 days air-liquid interface culture and negative control. Virtual serial sections of Calu-3 cells grown on the 3 μm membranes obtained by laser scanning microscopy as well as stained cryosections of cells on membranes confirmed that cells formed a monolayer and grew only on one side of the membrane (Fig D in S1 File). Similar to data by Zhang et al. [38] TEER values without cells (blank) did not differ between membranes with different pore sizes (125 ± 8 Ω*cm^2^ for 0.4 μm and 116 ± 8 Ω*cm^2^ for 3 μm).

### Aerosol generation devices

**Fig 2:**
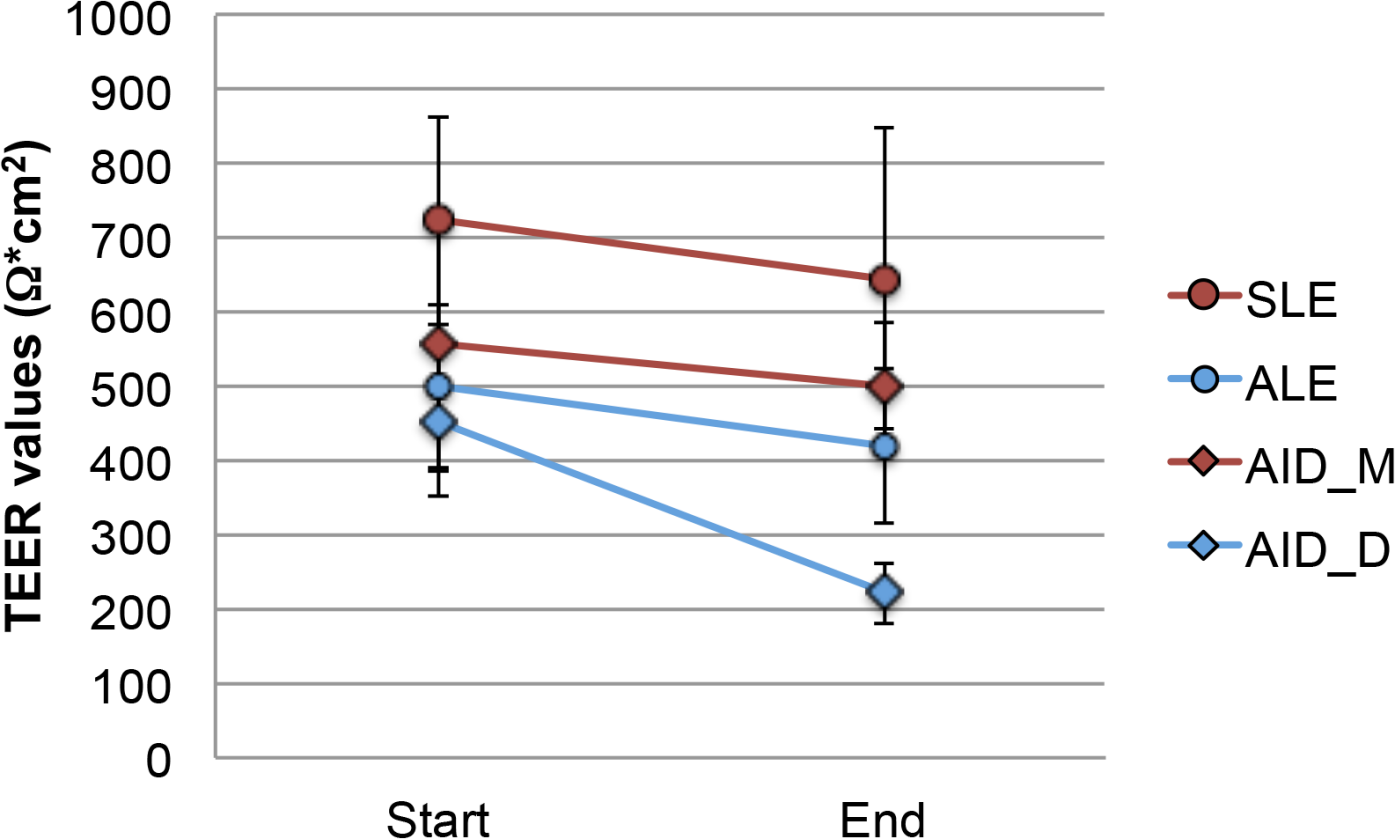
Differences in TEER values between start and end of the transport studies when fluorescein, rhodamine 123, budesonide and formoterol were applied as solution to cells grown in submersed culture (SLE) and at the air-liquid interface (ALE) or applied by MicroSprayer IA-1C Aerosolizer (AID_M) and by DP-4 Dry Powder Insufflator (AID_D) to cells grown at the air-liquid interface culture (n = 12). Significant decrease is marked by asterisk.

Aerosols generated by MicroSprayer IA-1C Aerosolizer were deposited with an efficacy of 27 ± 3%, as shown previously [21]. The application of aerosolized medium did not induce significant decreases in TEER values of Calu-3 cells cultured at an air-liquid interface (Fig 2).

The amounts of the compounds delivered by DP-4 Dry Powder Insufflator and applied as solution for ALE and SLE according to recovery at the end of the transport studies are listed in Table 2. Efficiency of delivery by DP-4 Dry Powder Insufflator was compound-specific and ranged from ~3% to 28%. A variable part of the powder did not leave the sample chamber. When the sample chamber was weighed before and after application around 50% of the initial dose for fluorescein, but only 10–20% of Symbicort powder had been retained. Moreover, powder was lost during the spraying and did not deposit on the cells. Total amounts of compounds applied as dry powder in the apical compartment (μg) were lower for fluorescein, rhodamine 123 and formoterol (alone and as Symbicort 160/4.5). Conversely, considerably higher amounts of budesonide were applied as dry powder than as solution. The calculated drug concentration (μg/ml) was in the same order of magnitude only for the fluorescent dyes, the concentrations of the budesonide and of formoterol alone were much higher when applied with DP-4 Dry Powder Insufflator than as solution. This caused different ratio of budesonide and formoterol (in μg delivered); in ALE and SLE the Bud/Form ratio was 1:10.9 compared to 27.7:1 in the Symbicort formulation applied by DP-4 Dry Powder Insufflator.

**Table 2:** Efficiency of delivery by DP-4 Dry Powder Insufflator (AID_D) and drug amounts in the apical compartment in ALE and SLE.

Compound	DP-4			Solutions	
	Efficiency of delivery (%)	Total amount (μg) ^#^	μg/ml*	Total amount (μg) ^#^	μg/ml
Fluorescein	13.7±4.4	1.4±0.2	27.5±4.4	4.9±0.2	10.1±0.2
Rhodamine 123	4.6±1.1	4.7±0.9	93.3±22.0	46.5±1.7	98.0±2.5
Budesonide	3.0±0.9	30.1±9.3	602.0±186.1	19.3±0.7	38.5±1.4
Budmix	23.5±2.6	47.0±5.2	940.7±103.0	9.04±1.1	18.1±2.3
Formoterol	9.6±1.9	96.5±19.8	1930.9±396.4	212.1±22.3	424.2±44.5
Formmix	28.2±1.1	1.7±0.1	33.9±1.3	104±4.5	207.9±9.1

*based on the recovered liquid in the apical chamber at the end of the AID exposures

^#^recovered amount. Abbreviations: Budmix, budesonide in combination with formoterol; Formmix, formoterol in combination with budesonide

Sizes of the particles delivered by the DP-4 Dry Powder Insufflator are formulation dependent and have to be determined experimentally [37]. The visualization by bright-field microscopy (Fig E in S1 File) indicated that, in general, powders were well dispersed with agglomerates preferentially seen for fluorescein. Delivered single particles of fluorescein (1.1±0.3 μm), budesonide (3.2± 1.3 μm), and Symbicort (6.9±1.4 μm) presented a roughly spherical shape and formed agglomerates. These agglomerates were largest (up to 18 μm) for Symbicort. Rhodamine 123 and formoterol deposited as elongated crystals of 8.6±1.9 x 14.7±3.8 μm and 9.1±3.5 x 17.1±1.2 μm, respectively. Larger agglomerates were rarely seen.

Cellular compatibility was assessed for the DP-4 Dry Powder Insufflator by using clean air as negative control in syringe-based dry powder aerosol exposure systems as suggested by Garcia-Canton et al. [39]. This exposure induced significant decreases of TEER values in the exposed cells (Fig 2). On the other hand, no decrease in cell viability according to bioreduction to formazan or release of lactic dehydrogenase as indication for plasma membrane damage was seen.

### Transport of dissolved compounds

When applied in solution P_app_ values of all compounds were higher upon ALE than upon SLE (Table 3). P_app_ values were highest for budesonide and lowest for the fluorescent dyes. Differences of P_app_ values between SLE and ALE were much less pronounced for budesonide.

**Table 3:** Overview of the influence of exposure method on P_app_ values for the different compounds (n = 4).

Compound	Exposure condition	P_app_ (10^−6^ cm/s)	Ratio P_app_ (ALE/ SLE)
Fluorescein	SLE	0.09±0.04	
	ALE	0.27±0.08	3.00
Rhodamine 123	SLE	0.07±0.02	
	ALE	0.27±0.09	3.86
Budesonide	SLE	13.18±0.06	
	ALE	17.93±1.36	1.36
Formoterol	SLE	1.72±0.24	
	ALE	2.98±0.79	1.73

**Fig 3:**
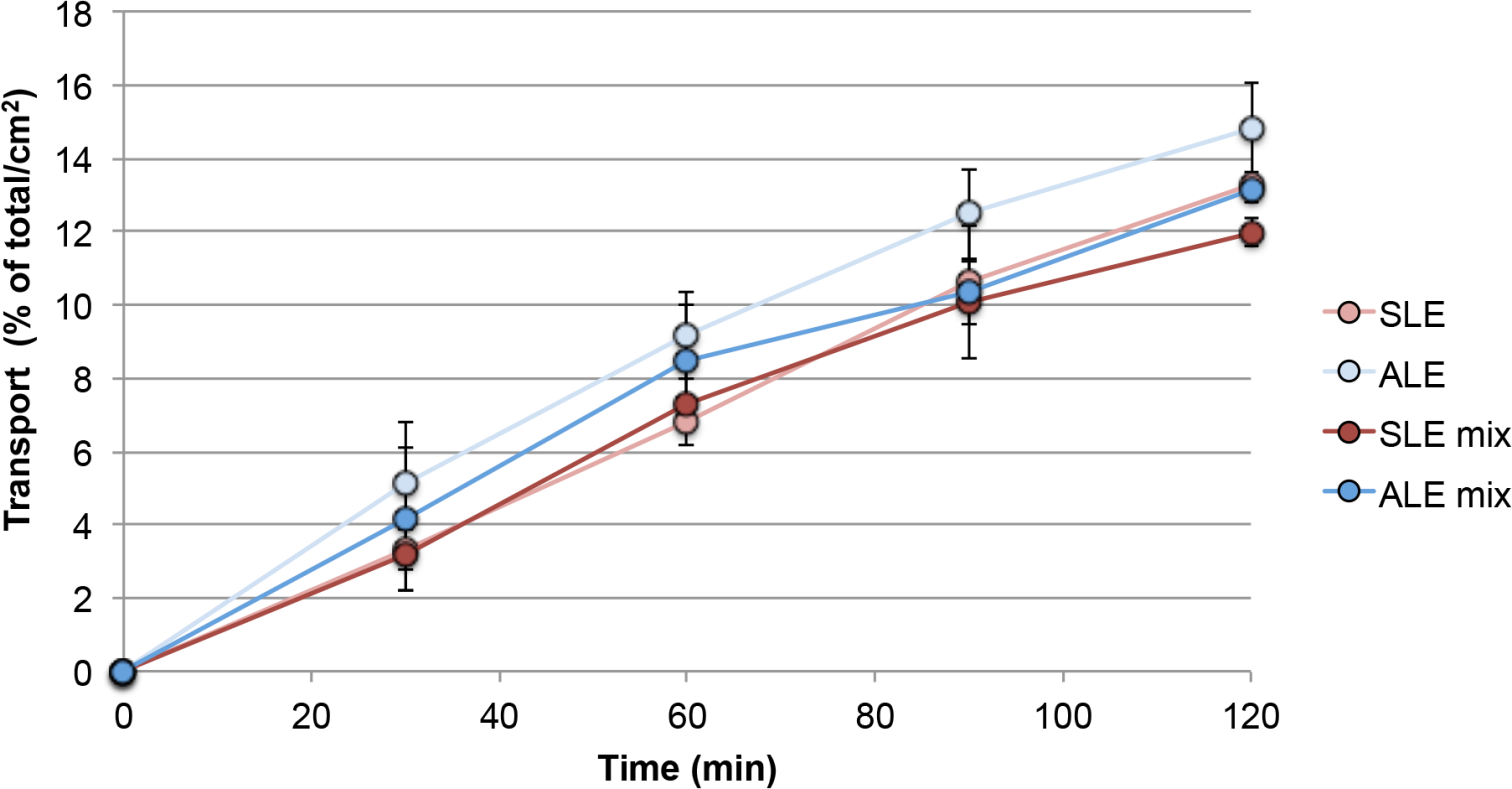
Transport rate of budesonide when applied in solution on cells in submersed culture (SLE) and on cells at an air-liquid interface (ALE). Abbreviations: SLE mix, ALE mix: mixture containing 50 μM budesonide and 0.5 mM formoterol fumarate was applied (n = 3). The total amount of drug applied to the cells was set as 100% and the transport rate was normalized to a membrane area of 1 cm^2^.

**Fig 4:**
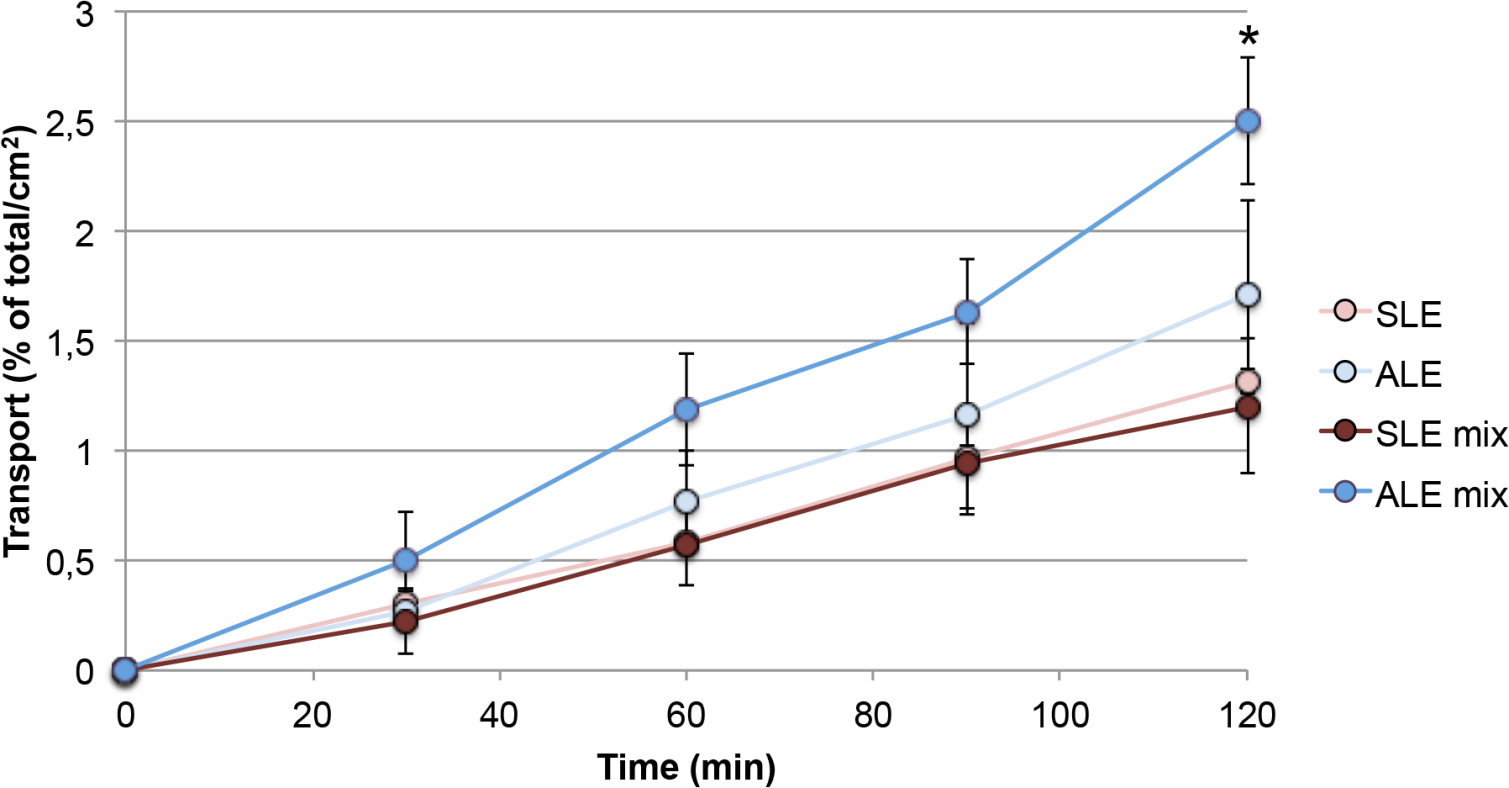
Transport rate of formoterol fumarate when applied in solution on cells in submersed culture (SLE) and on cells at an air-liquid interface (ALE). Abbreviations: SLE mix, ALE mix: mixture containing 50 μM budesonide and 0.5 mM formoterol fumarate was applied (n = 3). The total amount of drug applied to the cells was set as 100% and the transport rate normalized to a membrane area of 1 cm^2^. Significant effects are marked by asterisk.

Co-incubation of budesonide and formoterol did not show changes in budesonide transport neither in SLE nor in ALE (Fig 3). However, formoterol, which was transported only at low rates across the Calu-3 monolayers, showed higher transport rates when applied together with budesonide in ALE but not in SLE. The effect was significant after 2 hrs (Fig 4).

### Transport of aerosolized compounds

**Fig 5:**
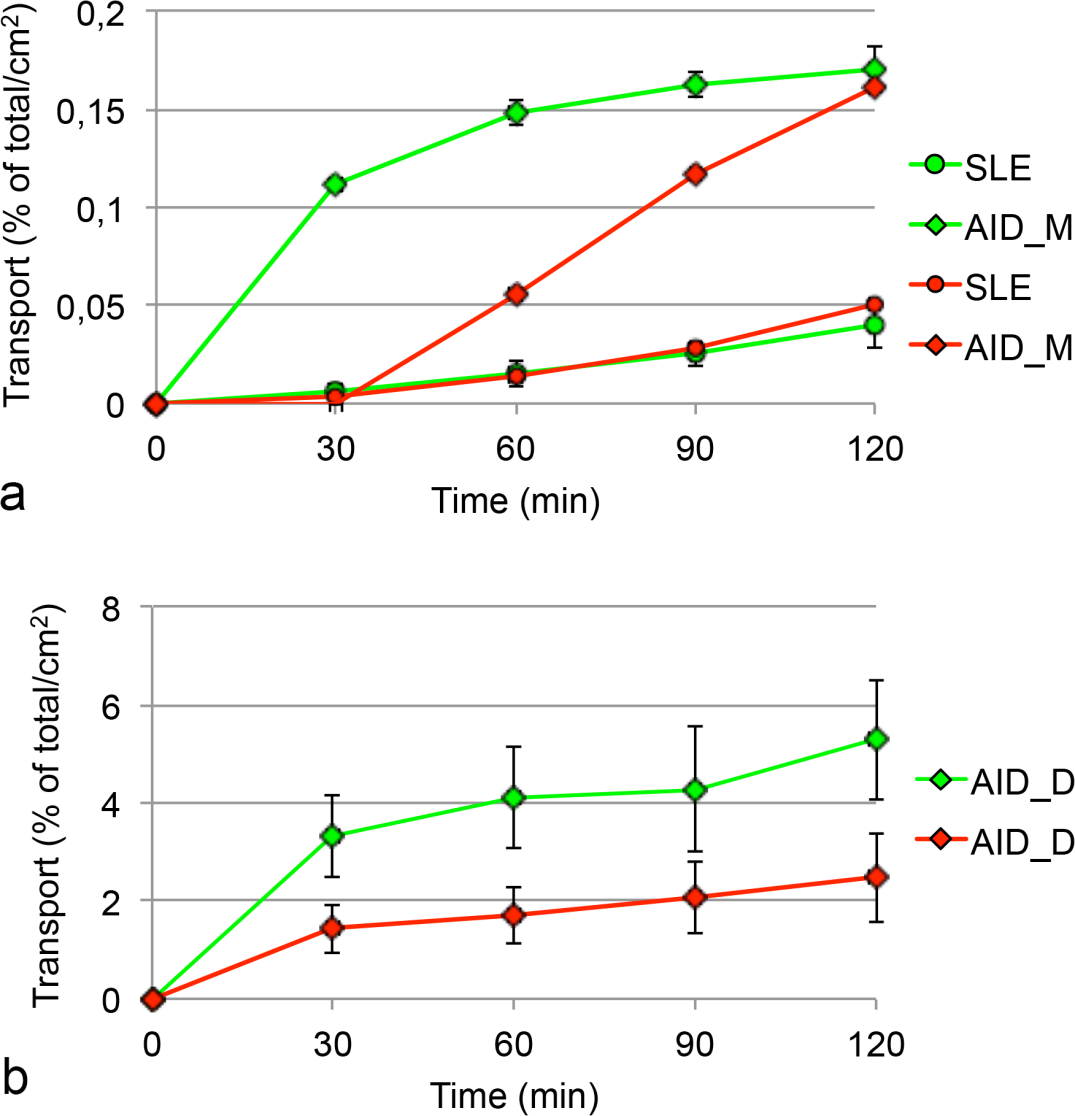
Transport of marker dyes across the Calu-3 monolayer (green, fluorescein; red, rhodamine 123) when applied as solution to cells grown in submersed culture (SLE) or applied with MicroSprayer IA-1C Aerosolizer (AID_M) and DP-4 Dry Powder Insufflator (AID_D) to cells cultured at the air-liquid interface (n = 6). The total amount of drug applied to the cells was set as 100% and the transport rate was normalized to a membrane area of 1 cm^2^.

Since these parameters are difficult to control the amount of recovered sample (start concentration in apical compartment * volume + amount in the samples + concentration in both compartments * volume) was set as 100%. The transport rates of the fluorescent dyes, fluorescein and rhodamine 123, applied by MicroSprayer IA-1C Aerosolizer was lower than after application by DP-4 Dry Powder Insufflator (Fig 5) and an initial delay in the transport (lag phase) was seen only upon application by MicroSprayer IA-1C Aerosolizer, (Fig 5A). Dye transport upon application with MicroSprayer IA-1C Aerosolizer was 4.3 times higher for fluorescein and 3.2 times higher for rhodamine 123 than upon SLE incubation. After application with the DP-4 Dry Powder Insufflator 131.7 times more fluorescein and 48.2 more rhodamine 123 were transported than upon SLE.

**Fig 6:**
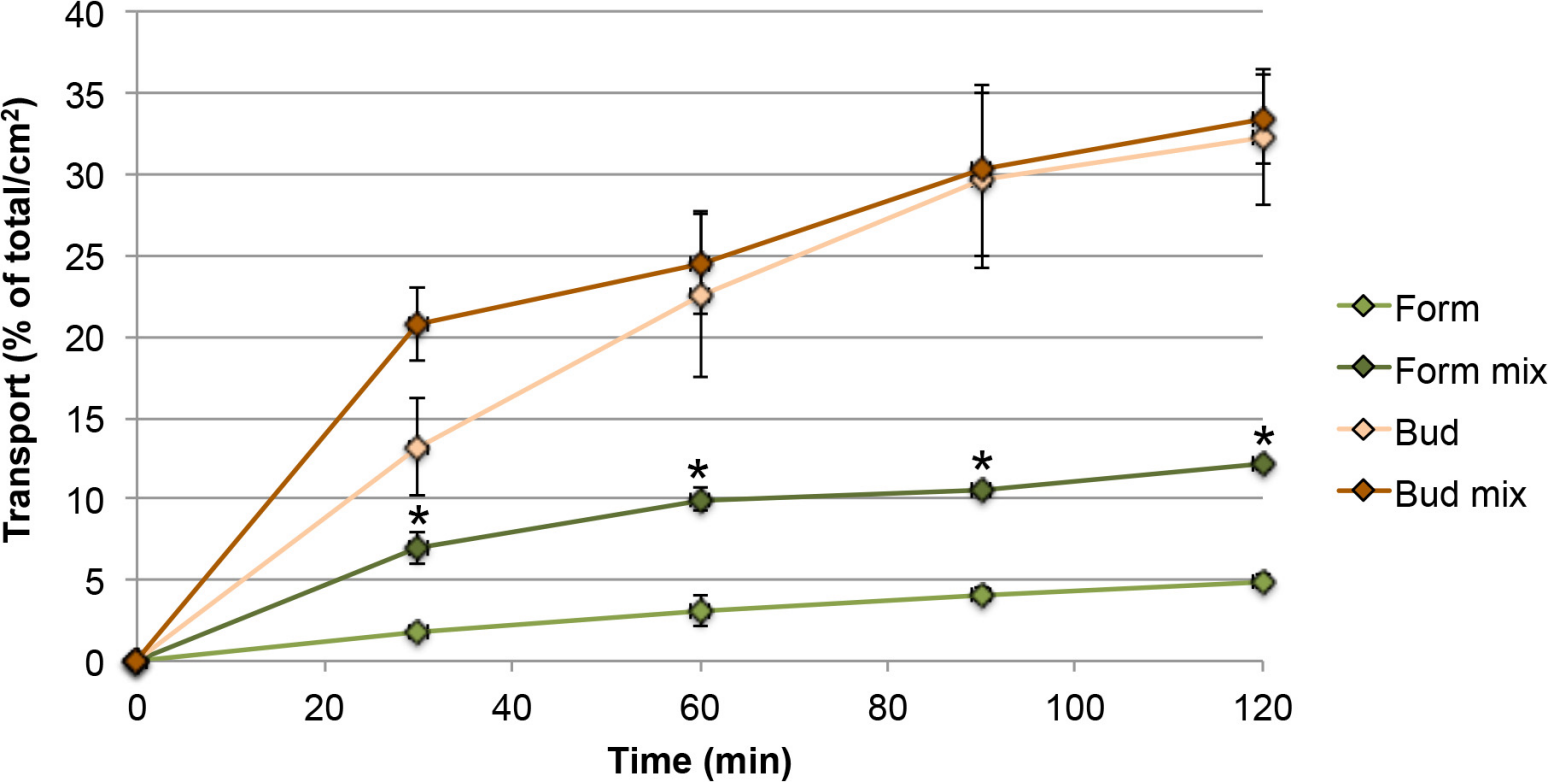
Transport of budesonide and formoterol fumarate as single substance and as combination product Symbicort in AID_D exposure. Abbreviations: Form mix: formoterol when applied as Symbicort powder; Bud mix: budesonide when applied as Symbicort (n = 3). The total amount of drug applied to the cells was set as 100% and the transport rate normalized to a membrane area of 1 cm^2^. Asterisks indicate differences between application as single substance and as combination.

Transport of formoterol in the fixed dose combination Symbicort was significantly higher than when applied alone by DP-4 Dry Powder Insufflator (Fig 6). For budesonide no differences between application alone and in combination with formoterol were seen.

## Discussion

This study demonstrated that, with some limitations, an exposure system consisting of manual devices for animal studies and Calu-3 cells in air-liquid interface culture could be used as screening tool for drug permeation in the preclinical assessment of formulations for oral inhalation.

### Cell culture

Similar to other studies, TEER values in this study were higher in cells cultured under submersed conditions (741 ± 101 Ω*cm^2^) than under air-liquid interface condition (494 ± 119 Ω*cm^2^). TEER values in submersed culture reported elsewhere ranged between 500–1200 Ω*cm^2^ [40–46], while they were 300–700 Ω*cm^2^ in air-liquid interface culture [45,47–52]. Consistent with data available in the literature [53] the membrane material did not influence TEER values in this study. Our Calu-3 cells also had similar TEER values when cultured on 3 μm pores and on 0.4 μm pores. This is in contrast to data published by Geys et al. who measured higher TEER values of Calu-3 cells on membranes with 0.4 μm pores than on membranes with 3 μm pores [46]. These differences, however, disappeared when cells were cultured for >5 days. The majority of studies [41,48,52], including this study, reported mucus production of Calu-3 cells only when cultured at an air-liquid interface, while only a minority of studies has demonstrated mucus production in Calu-3 cells in submersed culture [54,55]. Calu-3 cells have been switched from submersed culture to air-liquid interface culture after 24–48h in some studies and 2–5 days prior to the experiments in others [8,35,48,56–58]. Our comparison between 11d of air-liquid interface culture and 3 days of air-liquid interface culture showed that 3d were sufficient to induce the phenotype reached after 11 days based on TEER values and induction of mucus production. The short time to switch from submersed to air-liquid interface condition is not surprising given that transcriptional changes of protein expression are generally observed 48h after stimulation (e.g. [59]). Furthermore, human bronchial epithelial cells showed different basal levels of cytokines at three days of submersed and air-liquid interface culture [60].

### Aerosol generation device

As reported previously, delivery rates by the MicroSprayer IA-1C Aerosolizer in the selected set-up showed low dependency from the material that has been aerosolized; conventional compounds and nanoparticles in the size range from 20 nm to 200 nm showed similar deposition rates [29]. Aerosols generated from aqueous sodium fluorescein solutions were deposited with an efficacy of 27 ± 3%, of fluorescent polystyrene nanoparticles with an efficacy of 28±1.96% and of carbon nanotubes with an efficacy of 25±2.5% [21]. In contrast, delivery by DP-4 Dry Powder Insufflator showed considerable variations in delivery to the cells. These differences are linked to the properties of the aerosolized powders and are also seen in dry-powder inhalers. For instance, 80% of the metered dose in Symbicort Turbuhaler is delivered to the mouthpiece of the device. Particle size, shape, surface texture, contact area, surface energy, hygroscopy, relative humidity, and electrical properties are known to influence the delivery rate [61]. Furthermore, in our study also the distance between tip of the device and cells had an impact on aerosol delivery. It is known that emitted fractions of the DP-4 Dry Powder Insufflator are independent of the loaded amount but influenced by physicochemical properties of the formulation [37,62]. The observed differences are, therefore, not unexpected. Delivery of high amounts of APIs and potential cell damage by shear stress may cause concerns in the delivery by manual aerosol generation devices. For permeation studies traditionally relatively high drug concentrations are used in order to detect the drug in the basolateral compartment [63]. Due to the handling of the device even higher concentrations were used for the applications with DP-4 Dry Powder Insufflator. While no obvious cytotoxicity was seen by bioreduction to formazan and LDH release, TEER values were decreased when the DP-4 Dry Powder Insufflator was used. TEER values in these samples were below 300 Ω*cm^2^ and might not guarantee an intact barrier function of Calu-3 cells [64]. Choosing a greater distance between device and cells could prevent decreases of TEER values. However, the detection threshold of the compound in the basolateral compartment limits the distance between cells and device. This problem is particularly relevant when formulations contain drugs in highly different amounts and with different permeability. In this study the low amount of formoterol in Symbicort was the limiting factor for the distance between device and cells.

### Permeability and transport

P_app_ values of fluorescein reported in this study are similar to values published by other groups (0.1 x 10–6 cm/s for SLE and 0.1 x 10^−6^ cm/s or 0.94 x 10^−6^ cm/s for ALE [8,41,49,65–69]). The same applies for P_app_ values of rhodamine 123, which were reported as 0.49 x 10^−6^ cm/s in SLE and 2.27 x 10^−6^ cm/s for ALE [68,70]. The difference in the time-dependent permeation of drugs applied as solution (linear) and as aerosol (saturation) is consistent with the study by Bur et al. [35]. The non-linear transport curves suggest that sink conditions are not maintained. Although absorption of APIs in the lung usually is fast, non-sink conditions have also been suspected to occur in the deep lung [71,72]. Therefore, the culture model might mimic the situation in vivo where the small amount of lung lining fluid could be too low to completely dissolve the deposited particles.

The shape of the time-dependent transport curve for rhodamine 123 differed between the two aerosol applications, MicroSprayer IA-1C Aerosolizer and DP-4 Dry Powder Insufflator. While a lag phase in the transport was seen for application by MicroSprayer IA-1C Aerosolizer this delay was absent upon exposure by DP-4 Dry Powder Insufflator. Total amounts (in μg, Table 2) of rhodamine 123 were higher with the Aerosolizer (13.23 μg) than with Insufflator application (4.7 μg). Concentrations, on the other hand were similar (93.3 vs. 98.0 μg/ml). The delay in transport could be explained by the fact that rhodamine 123 is transported by MDR1/P-glycoprotein transporter [73]. The lack of delay upon application by DP-4 Dry Powder Insufflator might be caused by locally higher concentrations due to dissolution of the particles. In view of the observed decreases in TEER values in this exposure model, increased paracellular transport resulting from an impaired barrier function also cannot be excluded.

The effect of higher transport of formoterol across the monolayer in this study upon co-exposure with budesonide was observed when the compounds were applied as solution and as dry powder (Figs 4 and 6). It was specific for formoterol and could not be explained by differences in TEER values after application alone and in combination with budesonide. Single particles of Symbicort powder were smaller than formoterol particles but dissolution is very unlikely to be an important contributing factor because formoterol alone was used in a very high concentration. Interaction of budesonide with MRP1, MDR1/P-glycoprotein, OCT1, OCT2, and OCT3 and of formoterol with OCT3 and OCTN2 has been reported [74]. According to studies by Koepsell et al. budesonide can inhibit OCT2 activity [75], but this effect is not relevant in this model because Calu-3 cells do not express OCT2 [13]. Unexpected effects of OCT inhibitors (and high concentrations) on formoterol transport across Calu-3 layers have already been reported [13] and hypothesized to be linked to the bidirectionality of OCT mediated transport across the cell membrane.

As summarized in Table 4 the combination of Calu-3 cells and manual aerosol generation devices might provide more information on the interaction of different compounds on permeation. Main imitations of this study are that high powder concentrations of some APIs (budesonide) were used and that concentrations were not identical for all exposure models. The fact that similar findings were obtained in the different exposure conditions suggests that the differences in concentrations did not influence the observed effect. Studies for determination of P_app_ values in general use relatively high concentrations of APIs [63], and the observed effects on transporters might be different from effects at the lower concentrations invivo.

**Table 4:** Limitations and advantages of the manual devices for permeability testing.

Advantage	Limitation
Formulated (fixed dose) product can be tested to identify interaction between drugs using a relatively simple exposure system	The detection limit of one compound in the fixed dose combination limits the distance between DP-4 Dry Powder Insufflator and cells, and exposure can decrease TEER values of the exposed cells
Delivered aerosol is similar to in vivo experiments	Similar to intratracheal instillation in animals discrimination between the small particles that would reach the deep lung and larger particles is not possible
Different application forms (nebulized aerosols and dry powder aerosols) can be compared	Manual devices deliver different amounts of drugsAccuracy of the balance leads to application of non-physiologically high amounts of drugs

## Conclusions

Calu-3 cells are suitable for permeation studies because the desired phenotypes can be obtained largely independent from material and pore size of the transwell membranes and the duration of air-liquid interface culture when longer than 3 days. The use of manual aerosol generation devices for the assessment of transport and drug interaction at the cellular layer was more sensitive in identifying interactions of APIs but application of dry powders by DP-4 Dry Powder Insufflator presented problems due to compound-specific efficiency of delivery, high drug concentrations at the cell surface, and potential disruption of the cell monolayer. Conversely, MicroSprayer IA-1C Aerosolizer appears to be suitable for the testing of permeability of drug formulations for inhalation because TEER values did not decrease after the application and delivery was not compound-specific.

## Supporting Information

S1 File Contains Fig A in S1 File, Fig B in S1 File, Fig C in S1 File, Fig D in S1 File, Fig E in S1 File. (PDF)

S1 Table (PDF)

## References

[1] Mansour H, Xu Z, Meenach S, Park C, Rhee Y, DeLuca P (2015) Novel Drug Delivery Systems In: Mitra A, Kwatra D, Dutt Vadlapudi A, editors. Drug delivery. Burlington: Jones&Bartlett Learning pp. 71–107.

[2] Barnes PJ, Nicolini G, Bizzi A, Spinola M, Singh D (2012) Do inhaled corticosteroid/long-acting beta2-agonist fixed combinations provide superior clinical benefits compared with separate inhalers? A literature reappraisal. Allergy Asthma Proc 33: 140–144. 10.2500/aap.2012.33.3512 22525390

[3] Haghi M, Traini D, Postma DS, Bebawy M, Young PM (2013) Fluticasone uptake across Calu-3 cells is mediated by salmeterol when deposited as a combination powder inhaler. Respirology 18: 1197–1201. 10.1111/resp.12146 23796052

[4] Ehrhardt C, Laue M, Kim K (2008) In vitro Models of the Alveolar Epithelial Barrier In: Ehrhardt C, Kim K, editors. Drug Absorption Studies in Situ, in Vitro and in Silico Models. New York: Springer Science+Business Media LCC.

[5] Forbes B, Shah A, Martin GP, Lansley AB (2003) The human bronchial epithelial cell line 16HBE14o- as a model system of the airways for studying drug transport. Int J Pharm 257: 161–167. 12711171 10.1016/s0378-5173(03)00129-7

[6] Haghi M, Ong HX, Traini D, Young P (2014) Across the pulmonary epithelial barrier: Integration of physicochemical properties and human cell models to study pulmonary drug formulations. Pharmacol Ther. 10.1016/j.pharmthera.2014.05.003 24836727

[7] Foster KA, Avery ML, Yazdanian M, Audus KL (2000) Characterization of the Calu-3 cell line as a tool to screen pulmonary drug delivery. Int J Pharm 208: 1–11. 11064206 10.1016/s0378-5173(00)00452-x

[8] Mathias NR, Timoszyk J, Stetsko PI, Megill JR, Smith RL, Wall DA (2002) Permeability characteristics of calu-3 human bronchial epithelial cells: in vitro-in vivo correlation to predict lung absorption in rats. J Drug Target 10: 31–40. 11996084 10.1080/10611860290007504

[9] Haghi M, Salama R, Traini D, Bebawy M, Young PM (2012) Modification of disodium cromoglycate passage across lung epithelium in vitro via incorporation into polymeric microparticles. Aaps J 14: 79–86. 10.1208/s12248-011-9317-2 22203523 PMC3291191

[10] Sakamoto A, Matsumaru T, Yamamura N, Uchida Y, Tachikawa M, Ohtsuki S, et al (2013) Quantitative expression of human drug transporter proteins in lung tissues: analysis of regional, gender, and interindividual differences by liquid chromatography-tandem mass spectrometry. J Pharm Sci 102: 3395–3406. 10.1002/jps.23606 23670800

[11] Olsson B, Bondesson E, Borgström L, Edsbäcker S, Eirefelt S, Ekelund K, et al (2011) Pulmonary Drug Metabolism, Clearence, and Absorption In: Smyth H, Hickey A, editors. Controlled Pulmonary Drug Delivery. New York: Springer pp. 21–50.

[12] Bleasby K, Castle JC, Roberts CJ, Cheng C, Bailey WJ, Sina JF, et al (2006) Expression profiles of 50 xenobiotic transporter genes in humans and pre-clinical species: a resource for investigations into drug disposition. Xenobiotica 36: 963–988. 17118916 10.1080/00498250600861751

[13] Mukherjee M, Pritchard DI, Bosquillon C (2012) Evaluation of air-interfaced Calu-3 cell layers for investigation of inhaled drug interactions with organic cation transporters in vitro. Int J Pharm 426: 7–14. 10.1016/j.ijpharm.2011.12.036 22265910

[14] Sondergaard HB, Brodin B, Nielsen CU (2008) hPEPT1 is responsible for uptake and transport of Gly-Sar in the human bronchial airway epithelial cell-line Calu-3. Pflugers Arch 456: 611–622. 18094991 10.1007/s00424-007-0421-1

[15] Brillault J, De Castro WV, Couet W (2010) Relative contributions of active mediated transport and passive diffusion of fluoroquinolones with various lipophilicities in a Calu-3 lung epithelial cell model. Antimicrob Agents Chemother 54: 543–545. 10.1128/AAC.00733-09 19822706 PMC2798514

[16] Salomon JJ, Endter S, Tachon G, Falson F, Buckley ST, Ehrhardt C (2012) Transport of the fluorescent organic cation 4-(4-(dimethylamino)styryl)-N-methylpyridinium iodide (ASP+) in human respiratory epithelial cells. Eur J Pharm Biopharm 81: 351–359. 10.1016/j.ejpb.2012.03.001 22426135

[17] Bakand S, Hayes A (2010) Troubleshooting methods for toxicity testing of airborne chemicals in vitro. J Pharmacol Toxicol Methods 61: 76–85. 10.1016/j.vascn.2010.01.010 20117225

[18] Aufderheide M, Mohr U (2000) CULTEX—an alternative technique for cultivation and exposure of cells of the respiratory tract to airborne pollutants at the air/liquid interface. Exp Toxicol Pathol 52: 265–270. 10930128 10.1016/S0940-2993(00)80044-5

[19] Ritter D, Knebel JW, Aufderheide M (2003) Exposure of human lung cells to inhalable substances: a novel test strategy involving clean air exposure periods using whole diluted cigarette mainstream smoke. Inhal Toxicol 15: 67–84. 12476361 10.1080/08958370304449

[20] Lenz AG, Stoeger T, Cei D, Schmidmeir M, Semren N, Burgstaller G, et al (2014) Efficient bioactive delivery of aerosolized drugs to human pulmonary epithelial cells cultured in air-liquid interface conditions. Am J Respir Cell Mol Biol 51: 526–535. 10.1165/rcmb.2013-0479OC 24773184

[21] Fröhlich E, Bonstingl G, Hofler A, Meindl C, Leitinger G, Pieber TR, et al (2013) Comparison of two in vitro systems to assess cellular effects of nanoparticles-containing aerosols. Toxicol in Vitro 27: 409–417. 10.1016/j.tiv.2012.08.008 22906573 PMC3514486

[22] Fernandes CA, Vanbever R (2009) Preclinical models for pulmonary drug delivery. Expert Opin Drug Deliv 6: 1231–1245. 10.1517/17425240903241788 19852680

[23] Pritchard JN, Holmes A, Evans JC, Evans N, Evans RJ, Morgan A (1985) The distribution of dust in the rat lung following administration by inhalation and by single intratracheal instillation. Environ Res 36: 268–297. 3979359 10.1016/0013-9351(85)90025-8

[24] Mantecca P, Sancini G, Moschini E, Farina F, Gualtieri M, Rohr A, et al (2009) Lung toxicity induced by intratracheal instillation of size-fractionated tire particles. Toxicol Lett 189: 206–214. 10.1016/j.toxlet.2009.05.023 19501637

[25] Imanishi M, Dote T, Tsuji H, Tanida E, Yamadori E, Kono K (2009) Time-dependent changes of blood parameters and fluoride kinetics in rats after acute exposure to subtoxic hydrofluoric acid. J Occup Health 51: 287–293. 19483365 10.1539/joh.m8016

[26] Gervelas C, Serandour AL, Geiger S, Grillon G, Fritsch P, Taulelle C, et al (2007) Direct lung delivery of a dry powder formulation of DTPA with improved aerosolization properties: effect on lung and systemic decorporation of plutonium. J Control Release 118: 78–86. 17241685 10.1016/j.jconrel.2006.11.027

[27] Niwa Y, Hiura Y, Murayama T, Yokode M, Iwai N (2007) Nano-sized carbon black exposure exacerbates atherosclerosis in LDL-receptor knockout mice. Circ J 71: 1157–1161. 17587728 10.1253/circj.71.1157

[28] Witzenrath M, Gutbier B, Schmeck B, Tenor H, Seybold J, Kuelzer R, et al (2009) Phosphodiesterase 2 inhibition diminished acute lung injury in murine pneumococcal pneumonia. Crit Care Med 37: 584–590. 10.1097/CCM.0b013e3181959814 19114892

[29] Blank F, Rothen-Rutishauser BM, Schurch S, Gehr P (2006) An optimized in vitro model of the respiratory tract wall to study particle cell interactions. J Aerosol Med 19: 392–405. 17034314 10.1089/jam.2006.19.392

[30] Steiner S, Mueller L, Popovicheva OB, Raemy DO, Czerwinski J, Comte P, et al (2012) Cerium dioxide nanoparticles can interfere with the associated cellular mechanistic response to diesel exhaust exposure. Toxicol Lett 214: 218–225. 10.1016/j.toxlet.2012.08.026 22960666

[31] Sloane PA, Shastry S, Wilhelm A, Courville C, Tang LP, Backer K, et al (2012) A pharmacologic approach to acquired cystic fibrosis transmembrane conductance regulator dysfunction in smoking related lung disease. PLoS One 7: e39809 10.1371/journal.pone.0039809 22768130 PMC3387224

[32] Kardia E, Yusoff NM, Zakaria Z, Yahaya B (2014) Aerosol-based delivery of fibroblast cells for treatment of lung diseases. J Aerosol Med Pulm Drug Deliv 27: 30–34. 10.1089/jamp.2012.1020 23409833

[33] Heuking S, Rothen-Rutishauser B, Raemy DO, Gehr P, Borchard G (2013) Fate of TLR-1/TLR-2 agonist functionalised pDNA nanoparticles upon deposition at the human bronchial epithelium in vitro. J Nanobiotechnol 11: 29. 10.1186/1477-3155-11-29 PMC3765319 23964697

[34] Kim JS, Klosener J, Flor S, Peters TM, Ludewig G, Thorne PS, et al (2014) Toxicity assessment of air-delivered particle-bound polybrominated diphenyl ethers. Toxicology 317: 31–39. 10.1016/j.tox.2014.01.005 24451063 PMC3975599

[35] Bur M, Huwer H, Muys L, Lehr CM (2010) Drug transport across pulmonary epithelial cell monolayers: effects of particle size, apical liquid volume, and deposition technique. J Aerosol Med Pulm Drug Deliv 23: 119–127. 10.1089/jamp.2009.0757 20073555

[36] Teijeiro Osorio D (2007) Desarrollo de micro- y nanopartículas de polisacáridos para la administración pulmonar y nasal de macromoléculas terapéuticas Santiago de Compostela: Universidad de Santiago de Compostela. 391 p.

[37] Hoppentocht M, Hoste C, Hagedoorn P, Frijlink HW, de Boer AH (2014) In vitro evaluation of the DP-4M PennCentury insufflator. Eur J Pharm Biopharm 88: 153–159. 10.1016/j.ejpb.2014.06.014 24993307

[38] Zhang X, Zheng N, Zou P, Zhu H, Hinestroza JP, Rosania GR (2010) Cells on pores: a simulation-driven analysis of transcellular small molecule transport. Mol Pharmaceutics 7: 456–467. 10.1021/mp9001969 PMC2920490 20025248

[39] Garcia-Canton C, Errington G, Anadon A, Meredith C (2014) Characterisation of an aerosol exposure system to evaluate the genotoxicity of whole mainstream cigarette smoke using the in vitro γH2AX assay by high content screening. BMC Pharmacol Toxicol 15: 41 10.1186/2050-6511-15-41 25056295 PMC4122049

[40] Ren H, Suresh V (2014) A cell culture model for alveolar epithelial transport. PeerJ PrePrints 2: e256v251. 10.1371/journal.pone.0165225 PMC5079558 27780255

[41] Marusic M, Djurdjevic I, Draslar K, Caserman S (2014) Calu-3 model under AIC and LCC conditions and application for protein permeability studies. Acta Chim Slov 61: 100–109. 24664333

[42] Mamlouk M, Young PM, Bebawy M, Haghi M, Mamlouk S, Mulay V, et al (2013) Salbutamol sulfate absorption across Calu-3 bronchial epithelia cell monolayer is inhibited in the presence of common anionic NSAIDs. J Asthma 50: 334–341. 10.3109/02770903.2013.773518 23406450

[43] Harcourt JL, Haynes LM (2013) Establishing a liquid-covered culture of polarized human airway epithelial Calu-3 cells to study host cell response to respiratory pathogens in vitro. J Vis Exp. 10.3791/50157 PMC3600762 23426201

[44] Banga A, Witzmann FA, Petrache HI, Blazer-Yost BL (2012) Functional effects of nanoparticle exposure on Calu-3 airway epithelial cells. Cell Physiol Biochem 29: 197–212. 10.1159/000337601 22415089 PMC3711772

[45] Ehrhardt C, Fiegel J, Fuchs S, Abu-Dahab R, Schaefer UF, Hanes J, et al (2002) Drug absorption by the respiratory mucosa: cell culture models and particulate drug carriers. J Aerosol Med 15: 131–139. 12184863 10.1089/089426802320282257

[46] Geys J, Coenegrachts L, Vercammen J, Engelborghs Y, Nemmar A, Nemery B, et al (2006) In vitro study of the pulmonary translocation of nanoparticles: a preliminary study. Toxicol Lett 160: 218–226. 16137845 10.1016/j.toxlet.2005.07.005

[47] Madlova M, Bosquillon C, Asker D, Dolezal P, Forbes B (2009) In-vitro respiratory drug absorption models possess nominal functional P-glycoprotein activity. J Pharm Pharmacol 61: 293–301. 10.1211/jpp/61.03.0003 19222901

[48] Grainger CI, Greenwell LL, Lockley DJ, Martin GP, Forbes B (2006) Culture of Calu-3 cells at the air interface provides a representative model of the airway epithelial barrier. Pharm Res 23: 1482–1490. 16779708 10.1007/s11095-006-0255-0

[49] Fiegel J, Ehrhardt C, Schaefer U, Lehr C, Hanes J (2003) Large porous particle impingement on lung epithelial cell monolayers—Toward improved particle characterization in the lung. Pharm Res 20: 788–796. 12751635 10.1023/a:1023441804464

[50] Borchard G, Cassara ML, Roemele PE, Florea BI, Junginger HE (2002) Transport and local metabolism of budesonide and fluticasone propionate in a human bronchial epithelial cell line (Calu-3). J Pharm Sci 91: 1561–1567. 12115854 10.1002/jps.10151

[51] Florea BI, van der Sandt IC, Schrier SM, Kooiman K, Deryckere K, de Boer AG, et al (2001) Evidence of P-glycoprotein mediated apical to basolateral transport of flunisolide in human broncho-tracheal epithelial cells (Calu-3). Br J Pharmacol 134: 1555–1563. 11724763 10.1038/sj.bjp.0704390 PMC1573081

[52] Stentebjerg-Andersen A, Notlevsen IV, Brodin B, Nielsen CU (2011) Calu-3 cells grown under AIC and LCC conditions: implications for dipeptide uptake and transepithelial transport of substances. Eur J Pharm Biopharm 78: 19–26. 10.1016/j.ejpb.2010.12.030 21195173

[53] Dekali S, Gamez C, Kortulewski T, Blazy K, Rat P, Lacroix G (2014) Assessment of an in vitro model of pulmonary barrier to study the translocation of nanoparticles. Toxicology Reports 1: 157–171. 28962236 10.1016/j.toxrep.2014.03.003 PMC5598380

[54] Pezron I, Mitra R, Pal D, Mitra AK (2002) Insulin aggregation and asymmetric transport across human bronchial epithelial cell monolayers (Calu-3). J Pharm Sci 91: 1135–1146. 11948552 10.1002/jps.10114

[55] Patel J, Pal D, Vangal V, Gandhi M, Mitra AL (2002) Transport of HIV-protease inhibitors across 1 alpha,25di-hydroxy vitamin D3-treated Calu-3 cell monolayers: modulation of P-glycoprotein activity. Pharm Res 19: 1696–1703. 12458676 10.1023/a:1020761514471

[56] Garnett JP, Gray MA, Tarran R, Brodlie M, Ward C, Baker EH, et al (2013) Elevated paracellular glucose flux across cystic fibrosis airway epithelial monolayers is an important factor for Pseudomonas aeruginosa growth. PLoS One 8: e76283 10.1371/journal.pone.0076283 24124542 PMC3790714

[57] Bates E, Miller S, Alexander M, Mazur M, Fortenberry JA, Bebok Z, et al (2007) Bioelectric effects of quinine on polarized airway epithelial cells. J Cyst Fibros 6: 351–359. 17329172 10.1016/j.jcf.2007.01.001 PMC2077327

[58] Li Y, Wang W, Parker W, Clancy JP (2006) Adenosine regulation of cystic fibrosis transmembrane conductance regulator through prostenoids in airway epithelia. Am J Respir Cell Mol Biol 34: 600–608. 16399952 10.1165/rcmb.2005-0421OC PMC2644223

[59] Kim DS, Hubbard SL, Peraud A, Salhia B, Sakai K, Rutka JT (2004) Analysis of mammalian septin expression in human malignant brain tumors. Neoplasia 6: 168–178. 15140406 10.1593/neo.03310 PMC1502092

[60] Ghio AJ, Dailey LA, Soukup JM, Stonehuerner J, Richards JH, Devlin RB (2013) Growth of human bronchial epithelial cells at an air-liquid interface alters the response to particle exposure. Part Fibre Toxicol 10: 25 10.1186/1743-8977-10-25 23800224 PMC3750262

[61] Zeng X, Martin G, Marriott C (2001) Interparticulate Forces In: Zeng X, Martin G, Marriott C, editors. Particulate Interactions in Dry Powder Formulations for Inhalation. London: Taylor & Francis pp. 1–28.

[62] Duret C, Wauthoz N, Merlos R, Goole J, Maris C, Roland I, et al (2012) In vitro and in vivo evaluation of a dry powder endotracheal insufflator device for use in dose-dependent preclinical studies in mice. Eur J Pharm Biopharm 81: 627–634. 10.1016/j.ejpb.2012.04.004 22538097

[63] Artursson P, Karlsson J (1991) Passive absorption of drugs in Caco-2 cells In: Wilson G, Davis S, Illum L, Zweibaum A, editors. Pharmaceutical Applications of Cell and Tissue Culture to Drug Transport. New York: Plenum Press pp. 93–105.

[64] Steimer A, Haltner E, Lehr CM (2005) Cell culture models of the respiratory tract relevant to pulmonary drug delivery. J Aerosol Med 18: 137–182. 15966771 10.1089/jam.2005.18.137

[65] Tewes F, Paluch KJ, Tajber L, Gulati K, Kalantri D, Ehrhardt C, et al (2013) Steroid/mucokinetic hybrid nanoporous microparticles for pulmonary drug delivery. Eur J Pharm Biopharm 85: 604–613. 10.1016/j.ejpb.2013.03.020 23563102

[66] Grainger CI, Greenwell LL, Martin GP, Forbes B (2009) The permeability of large molecular weight solutes following particle delivery to air-interfaced cells that model the respiratory mucosa. Eur J Pharm Biopharm 71: 318–324. 10.1016/j.ejpb.2008.09.006 18845252

[67] Schulze C, Schäfer U, Voetz M, Wohlleben W, Venzago C, Lehr C (2011) Transport of metal oxide nanoparticles across Calu-3 monolayers modelling the air-blood barrier. EURO-NanoTox-Letters 1: 0001–0011.

[68] Haghi M, Young PM, Traini D, Jaiswal R, Gong J, Bebawy M (2010) Time- and passage-dependent characteristics of a Calu-3 respiratory epithelial cell model. Drug Dev Ind Pharm 36: 1207–1214. 10.3109/03639041003695113 20374185

[69] Gustafsson A, Lindstedt E, Elfsmark LS, Bucht A (2011) Lung exposure of titanium dioxide nanoparticles induces innate immune activation and long-lasting lymphocyte response in the Dark Agouti rat. J Immunotoxicol 8: 111–121. 10.3109/1547691X.2010.546382 21309687 PMC3104284

[70] Eixarch H, Haltner-Ukomadu E, Beisswenger C, Bock U (2010) Drug Delivery to the Lung: Permeability and Physicochemical Characteristics of Drugs as the Basis for a Pulmonary Biopharmaceutical Classification System (pBCS). J Epithel Biol Pharmacol 3: 1–14.

[71] Ruge CA, Kirch J, Lehr CM (2013) Pulmonary drug delivery: from generating aerosols to overcoming biological barriers-therapeutic possibilities and technological challenges. Lancet Respir Med 1: 402–413. 10.1016/S2213-2600(13)70072-9 24429205

[72] Sakagami M, Arora Lakhani D (2012) Understanding Dissolution in the Presence of Competing Cellular Uptake and Absorption in the Airways. Respiratory Drug Delivery 1: 185–192.

[73] Lee JS, Paull K, Alvarez M, Hose C, Monks A, Grever M, et al (1994) Rhodamine efflux patterns predict P-glycoprotein substrates in the National Cancer Institute drug screen. Mol Pharmacol 46: 627–638. 7969041

[74] Bosquillon C (2010) Drug transporters in the lung—do they play a role in the biopharmaceutics of inhaled drugs? J Pharm Sci 99: 2240–2255. 10.1002/jps.21995 19950388

[75] Koepsell H, Lips K, Volk C (2007) Polyspecific organic cation transporters: structure, function, physiological roles, and biopharmaceutical implications. Pharm Res 24: 1227–1251. 17473959 10.1007/s11095-007-9254-z

