# Supplementary material for: Permeation of Therapeutic Drugs in Different Formulations across the Airway Epithelium In Vitro
Source: PLoS One. 2015 Aug 14;10(8):e0135690. doi: 10.1371/journal.pone.0135690 (PMC4537286; doi:10.1371/journal.pone.0135690)
Supplement: S1 File — (PDF) [file pone.0135690.s001.pdf]

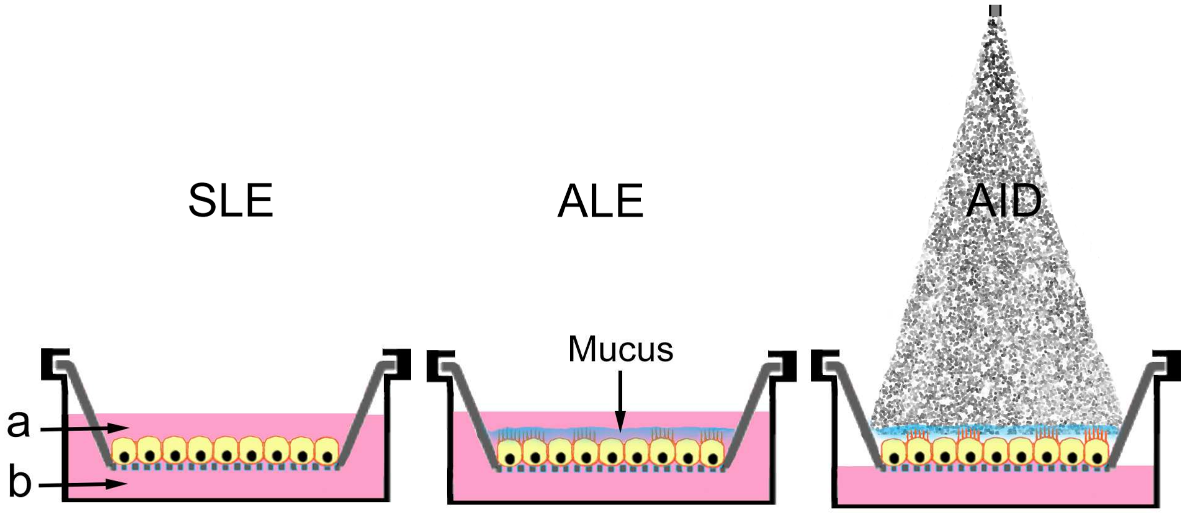

Fig. A: Exposure conditions: conventional submersed culture and liquid exposure (SLE), culture at the air-liquid interface and liquid exposure (ALE), air-liquid interface culture and aerosol exposure (AID).

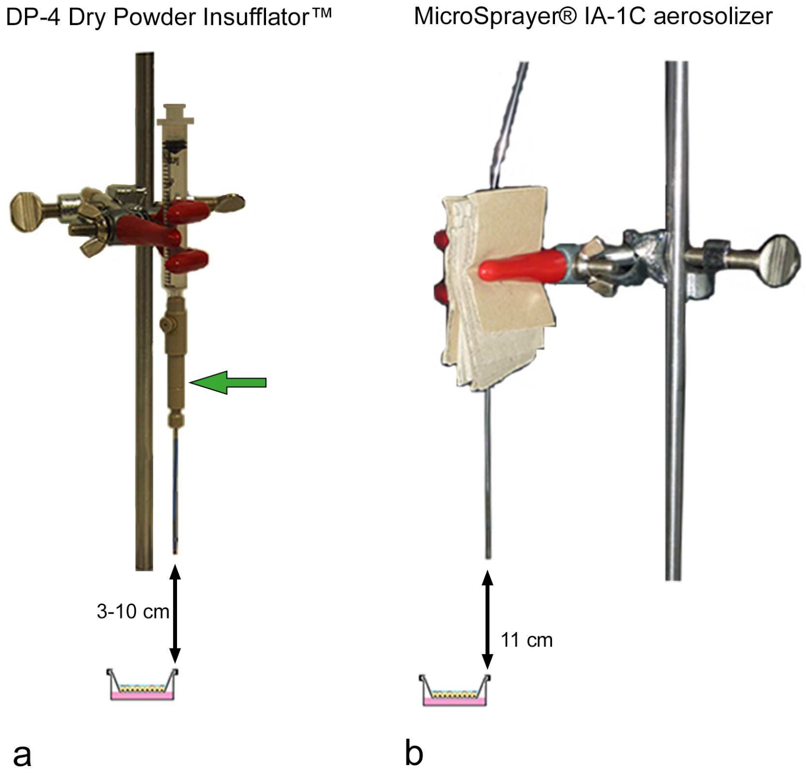

Fig. B: Exposure set-up using MicroSprayer® IA-1C aerosolizer (a) and DP-4 Dry Powder Insufflator™ (b). Experiments were performed with different distances between tip of the DP-4 Dry Powder Insufflator™ and rim of the cell culture plate. The distance for the applications with the MicroSprayer® IA-1C aerosolizer had been optimized in previous studies. Powder is added to the sample chamber (green arrow).

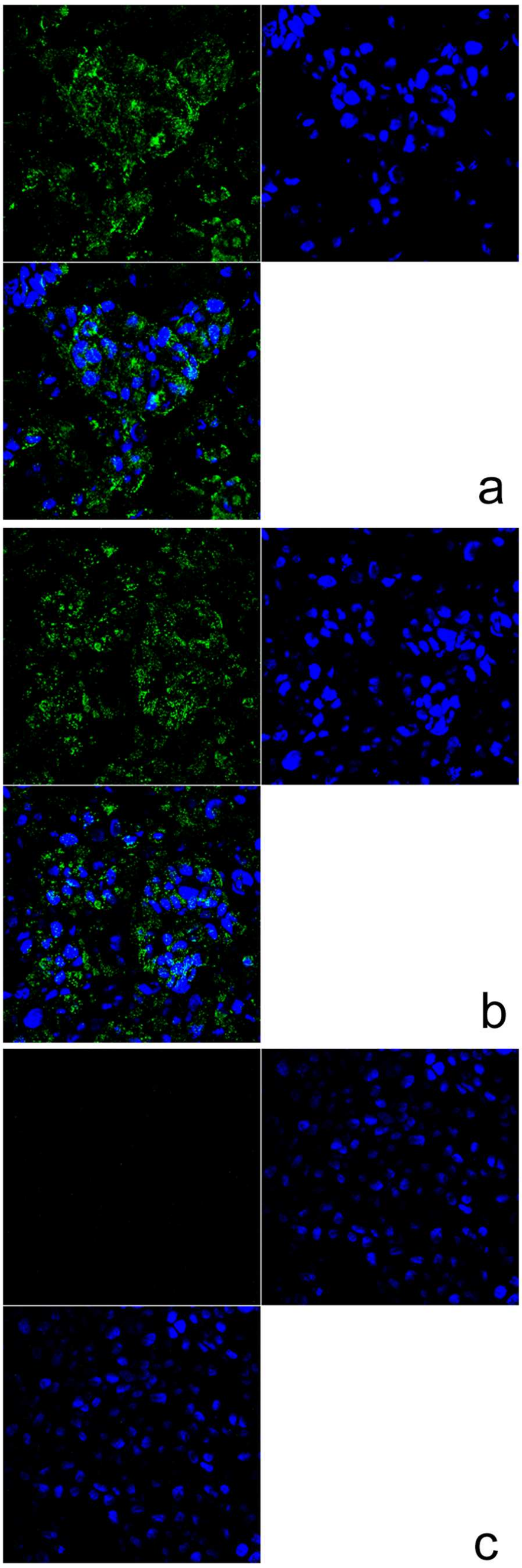

Fig. C: Examples of anti-mucin 5AC staining in Calu-3 cells cultured for 11 days (a) and for 3 days (b) at an air-liquid interface. Background staining (negative control) of the cells exposed to normal mouse IgG instead of the primary antibody is shown in c.

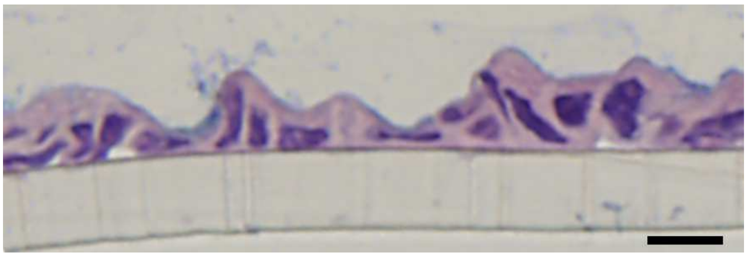

Fig. D: Cryosections of Calu-3 cells cultured at an air-liquid interface on 3 µm membrane inserts for 3 days prior to the experiments. Sections were stained with Alcian Blue and indicate mucus (light blue) at the apical pole of the cells. Scale bar: 10 µm.

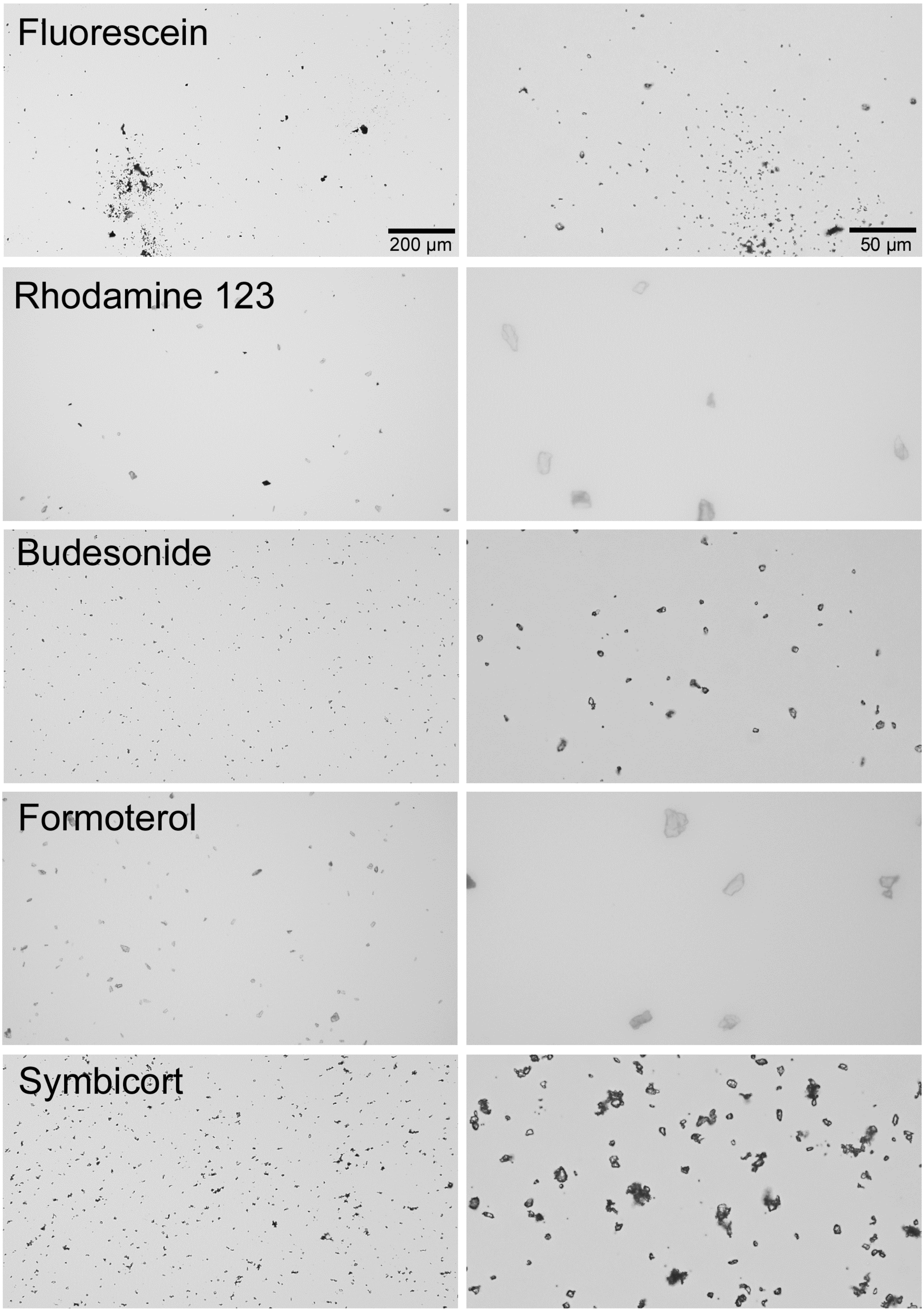

Fig. E: Brightfield images of powders produced by the DP-4 Dry Powder Insufflator™ device at different magnifications. Agglomeration of fluorescein particles is more obvious than for the other compounds. Fluorescein, budesonide and Symbicort particles display a more regular round shape, while rhodamine 123 and formoterol particles have different diameter to length ratios.
