# Supplementary material for: Permeation of Therapeutic Drugs in Different Formulations across the Airway Epithelium In Vitro
Source: PLoS One. 2015 Aug 14;10(8):e0135690. doi: 10.1371/journal.pone.0135690 (PMC4537286; doi:10.1371/journal.pone.0135690)
Supplement: S1 Table — (PDF) [file pone.0135690.s002.pdf]

Table 1s: Product information of tested membrane inserts

**Producer /trade name**	**Membrane material**	**Pore size (µm)**	**Pore density/cm²**	**Optical properties**	**Reference**
Corning® Transwell ®	Polycarbonate	0.4	1 x 10^8	Translucent	https://www.warneronline.com/Documents/uploader/Corning-Snapwell-Transwell%20Instruction%20Manual1.pdf
	Polyester	0.4	4 x 10^6	Clear	
	Polycarbonate	3	2 x 10^6	Translucent	
	Polyester	3	2 x 10^6	Clear	
Greiner Bio-One/ ThinCert ™	Polyethylene terephthalate	0.4	1 x 10^8	Translucent	http://www.greiner-bio-one.co.jp/products/PDF/ThinCertsPDF.pdf
	Polyethylene terephthalate	0.4	2 x 10^6	Transparent	
	Polyethylene terephthalate	3	2 x 10^6	Translucent	
	Polyethylene terephthalate	3	0.6 x 10^6	Transparent	
	Polycarbonate	3	2 x 10^6	Translucent	
